# Target Nanoparticles against Pancreatic Cancer: Fewer Side Effects in Therapy

**DOI:** 10.3390/life11111187

**Published:** 2021-11-05

**Authors:** Jorge A. Roacho-Pérez, Elsa N. Garza-Treviño, Paulina Delgado-Gonzalez, Zuca G-Buentello, Juan Luis Delgado-Gallegos, Christian Chapa-Gonzalez, Margarita Sánchez-Domínguez, Celia N. Sánchez-Domínguez, Jose Francisco Islas

**Affiliations:** 1 Departamento de Bioquímica y Medicina Molecular, Facultad de Medicina, Universidad Autónoma de Nuevo León, Monterrey 64460, Mexico; alberto.roachoprz@uanl.edu.mx (J.A.R.-P.); elsa.garzatr@uanl.edu.mx (E.N.G.-T.); paulina.delgadogn@uanl.edu.mx (P.D.-G.); zuca_95@hotmail.com (Z.G.-B.); juan_luisdg@hotmail.com (J.L.D.-G.); celia.sanchezdm@uanl.edu.mx (C.N.S.-D.); 2 Instituto de Ingeniería y Tecnología, Universidad Autónoma de Ciudad Juárez, Ciudad Juárez 32310, Mexico; christian.chapa@uacj.mx; 3 Grupo de Química Coloidal e Interfacial Aplicada a Nanomateriales y Formulaciones, Centro de Investigación en Materiales Avanzados, S.C. (CIMAV, S.C.), Unidad Monterrey, Apodaca 66628, Mexico; margarita.sanchez@cimav.edu.mx

**Keywords:** pancreatic cancer, molecular markers, target therapy, nanomedicine

## Abstract

Pancreatic cancer is the most common lethal tumor in America. This lethality is related to limited treatment options. Conventional treatments involve the non-specific use of chemotherapeutical agents such as 5-FU, capecitabine, gemcitabine, paclitaxel, cisplatin, oxaliplatin, or irinotecan, which produce several side effects. This review focuses on the use of targeted nanoparticles, such as metallic nanoparticles, polymeric nanoparticles, liposomes, micelles, and carbon nanotubes as an alternative to standard treatment for pancreatic cancer. The principal objective of nanoparticles is reduction of the side effects that conventional treatments produce, mostly because of their non-specificity. Several molecular markers of pancreatic cancer cells have been studied to target nanoparticles and improve current treatment. Therefore, properly functionalized nanoparticles with specific aptamers or antibodies can be used to recognize pancreatic cancer cells. Once cancer is recognized, these nanoparticles can attack the tumor by drug delivery, gene therapy, or hyperthermia.

## 1. Introduction

Cancer is one of the major causes of death in the world. In 2020, the prevalence of new cancer cases was approximately 19.3 million, and the prevalence of cancer deaths was 10 million. Thus, there is a prime interest in researching new ways to fight cancer [1]. Research over time has associated the use of chemotherapy for cancer with several adverse effects, including the limitation that, while it inhibits tumoral cell growth, chemotherapy also damages healthy cells in the process [2]. Over the years, the biochemical and molecular understanding of cancer and chemotherapy agents has led to new technologies for cancer treatment. Some of these arising technologies use the application of therapeutic nanoparticles [3,4].

**Figure 1:**
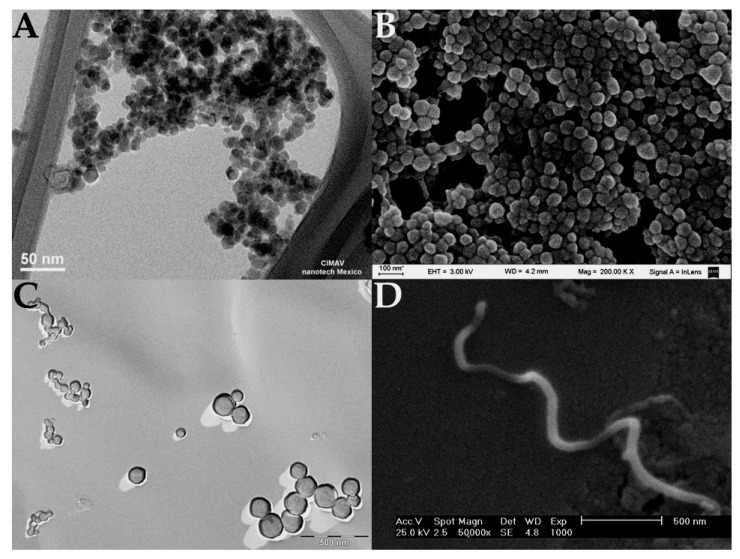
Electron microscopy images of different types of nanoparticles. (**A**) TEM image of magnetite nanoparticles [14], (**B**) SEM image of polymeric nanoparticles synthetized with bovine serum albumin (nanoparticles were coated with a gold layer), (**C**) TEM image of catanionic liposomes (image was obtained by cryofracture-TEM technique), and (**D**) SEM image of carbon nanotubes [15].

Nanotechnology can be described as the use of materials that have a diameter of 1–100 nm. Because of their substantially small size, nanomaterials may be formed by hundreds of millions of atoms [5]. Due to their high surface-to-volume ratio, the use of this type of carrier allows the delivery of small therapeutic biomolecules such as DNA [6], RNA [7], proteins [8], drugs [9], and other molecules to a specific tumoral site. Nanoparticles can be functionalized with some recognition molecules, such as antibodies [10] or aptamers [11], that can target the nanoparticle into the cancerous cells, avoiding the endocytosis into healthy cells. This targeting prevents toxicity to healthy cells and provides an efficient patient therapy [5]. Therefore, nanomedicine’s goal is to minimize adverse effects and enhance anticancer therapy. As shown in Figure 1, there are several types of nanoparticles, such as metallic nanoparticles, polymeric nanoparticles, liposomes, micelles, and carbon nanotubes [12]. Given their physicochemical and functional compositions, the properties of the nanoparticle may differ one from another, as it is shown in Table 1. The characteristics of the antineoplastic agent influence the design of the nanoparticle [13]. Hence, researchers worldwide have gained great interest in nanotechnology, as it may lead to better healthcare services and quality of life for cancer patients [12,13].

## 2. Pancreatic Cancer

Pancreatic cancer, one of the most aggressive of all oncological diagnoses, occurs more frequently between 60 and 80 years of age according to the latest update of GLOBOCAN (2020). It has an incidence of 495,773 cases worldwide with a mortality of 466,006. Latin America and North America together report 100,000 new cases (20.1% of all worldwide cases) and a mortality of 89, 307 cases (19.1%) [25].

### 2.1. Pancreatic Cancer Biology

The pancreas is considered a metabolic tissue because it is a gland positioned transversely on the posterior abdominal wall. Macroscopically, it is divided into head, body, and tail. Histologically, the pancreas has exocrine and endocrine functions. Acinar cells that produce digestive enzymes released into the small intestine represent its exocrine function. Its endocrine function includes β cells, from the islets of Langerhans, which produce insulin and α cells that produce glucagon. Insulin and glucagon are hormones responsible for maintaining optimum blood glucose levels [26]. Pancreatic cells can be affected by neoplasms. Cell alterations can lead to an incorrect production of necessary hormone levels, triggering diseases such as diabetes mellitus [27].

Pancreatic tumors are classified as either endocrine or non-endocrine; approximately 90% are sporadic and 10% hereditary [25]. Malignant tumors have different histological presentations—ductal adenocarcinoma (PDAC), which is the most frequent, cystadenocarcinoma, and other malignant tumors, such as sarcomas and metastases, that originate from another organ primary tumor [28]. Ductal adenocarcinoma lesions include

- Pancreatic intraepithelial neoplasms (PanIN), which are non-invasive microscopic lesions that occur in small pancreatic ducts (less than 0.5 cm).
- Intraductal papillary mucinous neoplasms (IPMN), precursor lesions of pancreatic cancer.
- Mucinous cystic neoplasms (MCN), which are also considered premalignant lesions of the pancreas and occur more frequently in women [29].

Pancreatic ductal adenocarcinoma has subtypes according to its morphology:

- Adenosquamous carcinoma, which has the worst prognosis.
- Mucinous carcinoma, with a favorable prognosis and is related to the lesion called intraductal papillary mucinous neoplasia.
- Undifferentiated anaplastic carcinoma, which is considered the most aggressive of the subtypes, with an extremely low survival rate due to its atypical cells mixed with osteoclast-like giant cells.
- Signet-ring-cell carcinoma, characterized by its invasive cells, and considered a rare form of pancreatic cancer [30,31].

### 2.2. Clinical Aspects of Pancreatic Cancer

The common risk factors for developing pancreatic cancer are smoking, obesity, poor diet quality, and a sedentary lifestyle. Smoking increases the risk of developing pancreatic cancer by 75% compared to non-smokers [32]. Another reported factor, which may suggest pancreatic cancer, is the appearance of diabetes mellitus particularly in patients older than 45 years [28]. The diagnosis of type 1 and 2 diabetes mellitus associates with a 1.8-times higher risk of developing pancreatic cancer, in Hispanic men [32]. A 5-year survival rate remains around 5–7% in all cases, and 1-year survival is reported in less than 20% of cases [33].

Genetically, there are multiple inherited disorders associated with the development of pancreatic cancer. Some genetic associated disorders can be Lynch syndrome, Peutz–Jeghers syndrome, familial adenomatous polyposis, Li–Fraumeni syndrome [33] and mutations of the following genes *PRSS1*, *KRAS*, *P16*, *P53*, and *BRCA2*. Overall, these genetic changes are considered high risk for developing pancreatic cancer [34].

Clinically, pancreatic cancer manifests with back pain, abdominal pain, diarrhea, and steatorrhea, all of which relate to poor lipid digestion in the absence of digestive enzymes. Other known manifestations are constipation, dyspepsia, nausea, vomiting, and involuntary weight loss. Interestingly, recent-onset jaundice has been described as a clinical finding that suggests malignancy in patients over 40 [35].

Albeit these manifestations are clear indicators, diagnosis continues as a challenge, as even though suspicion of pancreatic cancer can arise there continues to be the need for tumor confirmation. Endoscopic ultrasound has been shown to have a greater sensitivity to identify solid lesions of less than 2 cm as compared to secretin-enhanced magnetic resonance imaging and magnetic resonance cholangiopancreatography [31]. Moreover, multidetector computed tomography provides a broad anatomical coverage, allowing a complete view of local and distant disease, supporting its use in the diagnosis of suspected cancer [36].

### 2.3. Current Pancreatic Cancer Treatments

Pancreatic cancer lethality is, in part, related to poor treatment options. Most treatments involve the use of chemotherapeutical agents. Typically, chemotherapeutics are directed based on their efficiency, yet they continue to this day to have a potential drawback, as they are commonly associated with adverse side effects, as described on Table 2. Given these side effects, novel opportunities arise to improve pancreatic cancer treatment, most notably in the form of biomarkers which aid in targeting treatments and improving current therapies [37,38,39].

### 2.4. Surface Protein as Target in Pancreatic Cancer

As mentioned earlier, nanoparticles can be functionalized with antibodies or aptamers to focus the treatment only towards the cancerous cells and avoid being endocytosed by healthy cells. Pancreatic cancerous cells overexpress several surface proteins as compared to healthy cells (Table 3). Studies of surface targets in cancer have helped in recognizing “particular” antibodies, as well as aptamers immobilized over the nanoparticle’s surfaces, establishing a connection with nanoparticles and generating the endocytosis into cancerous cells. The overexpression of certain surface proteins, as presented on Table 3, in comparison of the normal expression in healthy cells, allows targeting of the treatment [51]. We should note that none of the presented surface proteins in Table 3, by themselves, are exclusive to pancreatic cancer, but certain group-expression is known to correlate directly with the presence of pancreatic cancer.

## 3. Nanoparticles as a Therapeutic Strategy in Cancer

Once nanoparticles are administered, they specifically focus and target cancer cells. These cells can then follow different strategies in order to be eliminated. The nature of the strategy depends on the design of the nanoparticle and the materials chosen for their construction. Some nanoparticles are made with highly biocompatible and biodegradable materials that function as a vehicle that carries a therapeutic agent [79]. These therapeutic agents can be a chemotherapeutics or biological molecules, such as a protein or a nucleic acid. Some other nanoparticles are built with specific materials such as metals, which produce heat or free radicals that eliminate cancer cells when they are excited with an external source of energy [80,81,82].

### 3.1. Nanoparticles for Drug Delivery

**Figure 2:**
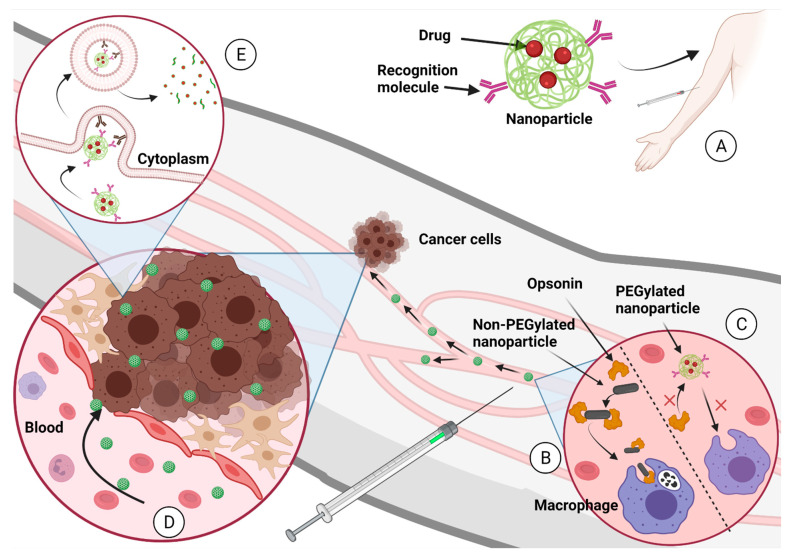
(**A**) The nanoparticle-carrying drugs are administered to the patient into the circulatory system. (**B**) Nanoparticles that are not designed properly can be eliminated by the macrophages after opsonization process. (**C**) Nanoparticles designed properly (PEGylated nanoparticles, for example) continue in blood circulation until they find the tumor. (**D**) The EPR effect propitiates the accumulation of nanoparticles in the tumor. (**E**) The recognition molecules over the nanoparticle surface target the membrane proteins from cancer cells and induce endocytosis. Once the nanoparticle is in the cancer cell cytoplasm, it degrades and delivers the chemotherapeutical to inhibit tumoral growth. Created with BioRender.com (accessed on 1 October 2021).

Chemotherapeutics can inhibit tumor growth or reduce metastasis. There are a lot of drugs that can be used as a cancer chemotherapeutic, but the problem remains as to whether these drugs are specific enough. Additional problems with chemotherapeutics include their poor aqueous solubility, non-specific distribution, fast elimination from blood circulation, and the development of drug resistance. To overcome these problems, modifications to the delivery has been seen as the best scenario. In Figure 2 we present how well-designed nanoparticle delivery strategies can help improve drug delivery [5,17,83].

Studies have shown that different drugs can be loaded into nanoparticles. Drugs used for pancreatic cancer treatment have been loaded in different nanoparticles: 5-FU in lipid nanocapsules [84], capecitabine and cisplatin in composite micelles [85], gemcitabine in polyhydroxy-butyrate-coated magnetic nanoparticles [86], oxaliplatin in a long-circulating thermosensitive smart-release liposome [87], and irinotecan in pH-sensitive and peptide-modified liposomes and solid lipid nanoparticles [88]. Drugs can be attached to nanoparticles by creating a covalent or non-covalent bond. Nanoparticles can be loaded with two or more drugs for simultaneous administration, potentiating a synergistic therapeutic effect [85]. Nanoparticles can be designed to be hydrophobic, hydrophilic, or amphipathic, increasing the solubility of the hydrophobic drug in blood plasma. A drug carried by a nanoparticle has a prolonged blood circulation time because the drug is not easily degraded by enzymes or eliminated by the immune system [5,17,83]. Macrophages carry out elimination of the particles in the immune system. Opsonin proteins from plasma typically can cover the nanoparticles, inducing the macrophages to recognize and eliminate the nanoparticle from the blood. A strategy to avoid phagocytosis by macrophages is to functionalize the nanoparticle with the biocompatible and non-immunogenic hydrophilic polymer polyethylene glycol (PEG). This functionalization avoids the mobilization of opsonins over the nanoparticle surface. The long circulation time improves drug distribution across the whole body [89,90,91]. In addition, nanoparticles can cross membranes and epithelial layers because of their physical characteristics. Another reported phenomenon is the accumulation of nanomedicines into tumors. This phenomenon is known as the enhanced permeability and retention (EPR) effect. This effect occurs because most solid tumors have blood vessels with defective architecture, which provides better vascular permeability to ensure a sufficient supply of nutrients and oxygen to the tumor for its proliferation. If the nanoparticle is functionalized with a recognition molecule, such as an aptamer or an antibody, it can be endocytosed by the cancer cell. Nanomedicine’s goal in drug delivery is to target the nanoparticle and deliver the chemotherapeutic into the cancer cell to decrease cytotoxicity in healthy cells [5,17,83].

### 3.2. Nanoparticles as a Vehicle for DNA (Gene Therapy)

For cancer treatment, some nanoparticles are used as a vehicle for the delivery of DNA. This DNA can contain a gene sequence that expresses a protein than can “fix” the cancerous cell. However, the most-studied strategy is the administration of DNA that contains the sequence of a suicide gene that expresses a lethal protein that “kills” the cancerous cell. The killer proteins are proteins that induce apoptosis or necrosis in cancer cells [92].

**Figure 3:**
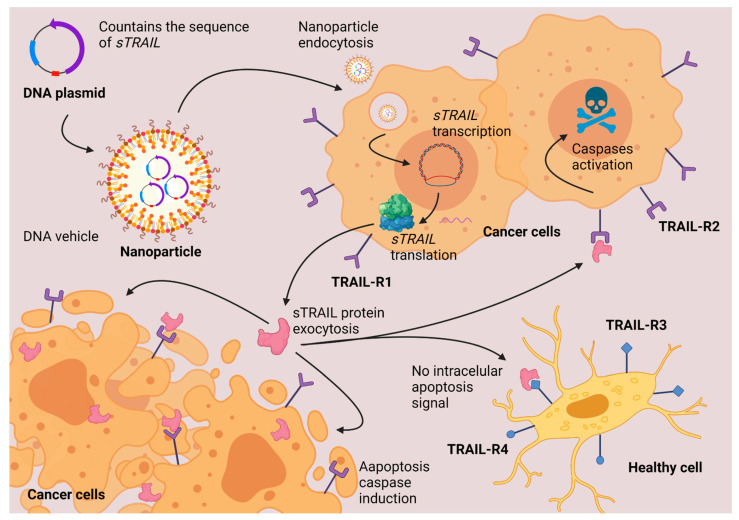
TRAIL gene therapy. Nanoparticles deliver the DNA inside the cells. The cells synthesize a soluble fraction of TRAIL protein. TRAIL induces apoptosis via caspases activation only in cancerous cells that overexpress TRAIL-R1 and TRAIL-R2 protein-membrane receptors. Created with BioRender.com (accessed on 1 October 2021).

A recent example of successful of targeting cancer cells has been described with particles delivering genes of the TNF superfamily. Protein expression of TNFα and DC95 has given good results causing necrosis in cancerous cells [3,52]. Another protein molecule from the same family is TNF-related apoptosis-inducing ligand (also known as TRAIL or TNFSF10) which is known to cause the death of cancerous cells without presenting secondary effects in the patient. Nanoparticles delivering plasmid DNA with the sequence of a suicide gene such as *TRAIL*, express the protein that causes apoptosis preferentially in cancer cells without affecting the healthy tissues, as shown in Figure 3. TRAIL protein is a transmembranal protein. Some proteases that involve cysteine protease activity can release the soluble fraction of TRAIL (sTRAIL) to the plasma. In an adult individual, the concentration of sTRAIL is approximately 100 pg/mL. At this concentration, the sTRAIL can induce apoptosis in most of the cell lines in vitro. The induction of apoptosis begins with the union of TRAIL with a specific receptor. TRAIL can bind to four different membrane receptors. When TRAIL binds with TRAIL-R1 or with TRAIL-R2, there is an induction of apoptosis. When TRAIL binds to TRAIL-R3 or TRAIL-R4, apoptosis truncates, and the apoptotic effect of TRAIL is stopped. All the TRAIL receptors are transmembranal proteins; TRAIL-R1 and TRAIL-R2 have an intracellular death domain (DD) responsible for inducing apoptosis. TRAIL-R3 lacks an intracellular domain, which is why there is no apoptosis induction. TRAIL-R4 induces other cellular pathways different from apoptosis (NF-κB activation). The apoptosis activated by TRAIL-R1 and TRAIL-R2 is mediated by the activation of caspases, principally caspase 3. TRAIL has provided good results in preclinical studies in mice as a cancer therapeutic against cancer cells which overexpress TRAIL pro- apoptotic receptors. There are still some challenges in developing the half-time circulation in blood and delivery in targeted cells. Some authors propose the use of nanoparticles to improve the delivery of TRAIL [93,94,95,96].

### 3.3. Nanoparticles as a Vehicle for RNAi (Gene Therapy)

**Figure 4:**
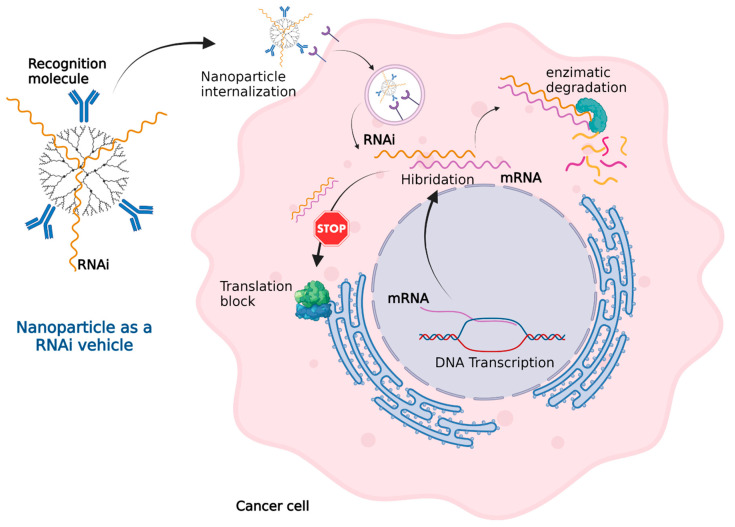
Nanoparticles as an RNAi vehicle. The RNAi–mRNA complex structure can be recognized by degradation enzymes and cannot be read by the ribosomes for translation. Created with BioRender.com (accessed on 1 October 2021).

In cancer cells, some DNA sequences such as oncogenes, chromosomal rearrangements, insertion mutations, point mutations, and gene amplification express messenger RNA (mRNA) that generate a cancerous phenotype. RNA interference (RNAi) technology, as shown in Figure 4, can effectively inactivate mRNA. Nanoparticles can administer RNAi into cancer cells in a similar fashion to how they deliver other genetic material.

The RNAi sequence is designed to be complementary with the mRNA that needs to be inactivated. The mRNA from the cancer cell generates a complex with the synthetic RNAi. This mRNA–RNAi complex cannot be read by the ribosomes blocking the translation or even recognized by enzymes leading complex degradation. The mRNA inactivation leads to the inhibition of tumoral growth, invasion, or migration. RNAi technology in combination with traditional chemotherapy can improve the treatment of cancer [21,97,98,99].

Targeted therapies that directly block specific oncogenic pathways in PDAC progression have so far played a limited role in the treatment of this disease. Multiple signaling pathways are affected in pancreatic cancer and can serve as therapeutic targets. Some approaches have focused on the main genes that are associated with the initiation, maintenance, and progression of PDAC, such as the common mutations on *KRAS* (>90% of all the PDAC cases), *TP53* (64%), *CDKN2A* (17%), and *SMAD4* (21%), which are mutated in a large percentage of patients with this type of cancer [100,101]. In 2019, Mehta evaluated bovine serum albumin nanoparticles for the delivery of RNAi targeting *KRAS* G12S mutation [52]. *KRAS* is activated when linked to GTP and deactivated when linked to GDP. The intrinsic cycle of *KRAS* GTP–GDP is regulated by guanine-nucleotide-exchange factors (GEF) that stimulate nucleotide exchange and GTPase-activating proteins (GAP) that accelerate the intrinsic hydrolysis activity of KRAS GTP. *KRAS* was the first candidate target to treat PDAC. These mutations have therapeutic implications, especially since the targets are multiple, whether at the genetic level per se, during their post-translational maturation, in the interaction with nucleotides, or after the activation of the nucleotides. Once the *KRAS* protein is bound to GTP, it interacts with over 80 downstream effector proteins and signaling pathways, such as mitogen-activated protein kinase (MAPK), MAPK kinase (MEK), phosphoinositide 3-kinase (PI3K), AKT, the mechanistic target of rapamycin (mTOR) or rapidly accelerated fibrosarcoma (RAF), or extracellular signal-regulated kinase (ERK). Each of these *KRAS* effectors has been proposed as a therapeutic target to regulate PDAC progression. In addition, targeted therapies that the United States Food and Drug Administration (FDA) has approved as treatments for pancreatic cancer include epidermal growth factor receptor (EGFR/ErbB) inhibitors and tyrosine kinase inhibitor (TKI) [100,101].

On the other hand, in vivo administration of nucleic acids (DNA or RNA) is still a challenge due to short blood circulation. Enzymes degrade nucleic acids delivered directly in blood. Different materials are used for the construction of nanoparticles for nucleic acid delivery. Cationic charged polymers are used for carrying the anionic charged nucleic acids [20]. Polyethylenimine (PEI) shows high in vitro transfection efficiency, but it has a lot of problems in in vivo administration because of toxic behavior and a lack of stability [102]. An alternative is to conjugate different materials to improve their deficiencies. For example, PEI can be conjugated with PEG to reduce PEI toxicity [103]. Other cationic polymers that can be used are poly-L-lysine (PLL) [104], chitosan, hyaluronic acid [105], alginate [106], and poly(lactic-co-glycolic acid) (PLGA) [107]. Another cationic material that can be used for nanoparticle synthesis is lipids. They can form liposomes, micelles, emulsions, or solid lipid nanoparticles [108]. Some inorganic substances also can be used for nucleic acid delivery, such as mesoporous silica nanoparticles [109], carbon nanotubes [110], and metallic nanoparticles [111]. Inorganic materials can also be combined with cationic polymeric materials to improve their proprieties [20].

### 3.4. Nanoparticles for Photothermal Therapy

**Figure 5:**
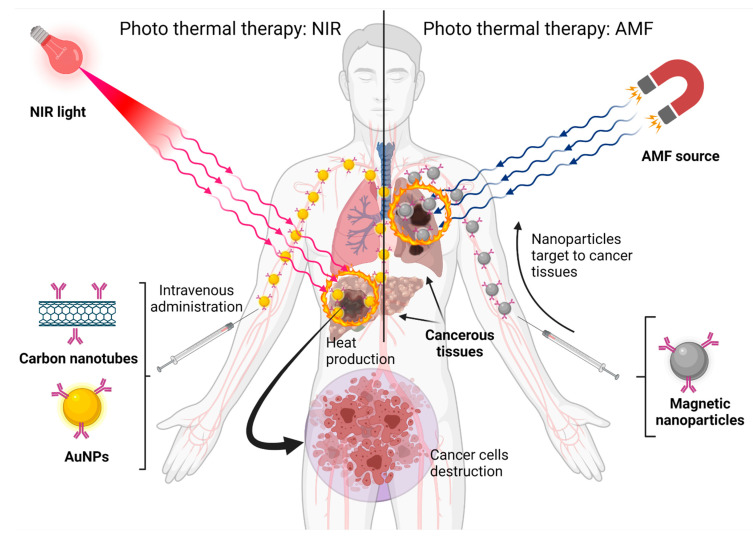
Nanoparticles built with different materials can be exposed to different energy sources and produce heat to eliminate cancer cells. Created with BioRender.com (accessed on 1 October 2021).

Some nanomaterials, such as gold nanoparticles and carbon nanotubes, can absorb near-infrared (NIR) light at 650–900 nm and convert it to heat. Tissues poorly absorb NIR light, so it is not dangerous. Other materials, such as magnetic materials, can generate heat when exposed to an alternating magnetic field (AMF). This heat cannot hurt healthy cells, but tumor cells are heat-sensitive. As is shown in Figure 5, the heat produced by nanoparticles can destroy cancer cells by eliminating tumors and suppressing distant metastasis. Photothermal therapy, in combination with chemotherapy and radiation, can improve cancer therapeutic outcomes [5,17]. If carbon nanotubes are used in this therapy, they need to be combined with other materials to avoid problems associated with the use of carbon nanotubes—poor solubility in water, low biodegradability and dispersity, toxicity problems, and possible effects in the proteome and genome [24]. Another variation of this therapy is the photodynamic therapy, which needs molecular oxygen (O_2_). The nanoparticle exposed to the light generates photodynamic reactions that eliminate cancerous cells without causing harm to healthy cells [112].

## 4. Conclusions

Although pancreatic cancer is one of the deadliest cancers, when a search of under-development treatments is performed on databases, there is less information in comparison with other kinds of cancer. Because of the biological nature of pancreatic cancer, there are surface proteins that are overexpressed in cancer cells in comparison with healthy cells. Using nanoparticles, functionalized with antibodies or aptamers, it is possible to develop targeted treatments against these molecular targets. Using nanoparticles, the treatment can be the targeted administration of a conventional chemotherapeutic (5-FU, capecitabine, cisplatin, gemcitabine, oxaliplatin, or irinotecan), or the administration of novel molecules such as RNAi or suicide DNA genes. Another promising technology that implicates the use of nanoparticles and produces fewer side effects than conventional therapies, is the development of photothermal and photodynamic therapies. This nanotechnology could revolutionize the actual treatments against pancreatic cancer. However, the main challenges in nanomedicine relate to the development of pre-clinical models which can translate to human pancreatic cancer, the effective and efficient assessment of pancreatic cancer therapy with nanoparticles and chemo-therapeutic combinations in early-phase clinical studies, and the development of improved regulatory endpoints for pancreatic cancer nanomedicine.

## Figures and Tables

**Table 1:** Properties of the nanoparticles.

Nanoparticle	Unique Properties	Medical Use in Cancer	References
Magnetic nanoparticles (MNPs)	Can be detected and manipulated by remote magnetic fields, can generate heat when exposed to an alternating magnetic field.	Magnetic biosensing, magnetic imaging, and magnetic separation (diagnostics). Drug and gene delivery, and hyperthermia therapy.	[5,16,17]
Gold nanoparticles (AuNPs)	Surface plasmon resonance, surface multi-functionalization, facile synthesis, stable nature, non-toxic and non-immunogenic nature, high permeability and retention effect, easy penetration and accumulation at tumor, can absorb near-infrared (NIR) light at 650–900 nm and convert it to heat.	Tumor detection by imaging (diagnostics). Treatment of cancer by drug delivery, photothermal and photodynamic therapy.	[5,17,18]
Polymeric nanoparticles	Biodegradable, increase the circulation time of drugs in the body, can target molecules with minimal side-effects, non-activation of the mononuclear phagocyte system.	Polymeric nanocarrier system for drug delivery in chemotherapy. Cationic charged polymers can carry nucleic acids (gene therapy). Controlled drug delivery.	[19,20]
Liposomes	Biocompatible, highly flexible, can carry different types of therapeutic molecules, can be tailored to extend blood circulation time, can be targeted. Several liposomes (lipidic nanoparticles) are on the market.	Drug delivery, long-circulating (PEGylated) liposomes, gene therapy, ligand-targeted liposomes, liposomes containing combinations of drugs. Delivery of anti-fungal, antibiotic, anesthetic, and anti-inflammatory drugs.	[21,22]
Micelles	Self-assembly, condensation and protection of nucleic acids, cell association, gene transfection, low toxicity.	Gene delivery	[23]
Carbon nanotubes (CNTs)	Can penetrate cell membranes, the sp2 hybridization of all carbons enables their functionalization with almost every biomolecule or compound, allowing them to target cells and deliver drugs under the appropriate environmental stimuli, can absorb near-infrared (NIR) light at 650–900 nm and convert it to heat.	Drug delivery and hyperthermia therapy.	[24]

**Table 2:** Actual pancreatic cancer chemotherapy and their side effects.

Drug	Action Pathway	Common Adverse Side Effects (>30%)	Less Common Adverse Side Effects (<30%)	References
5-FU Capecitabine Gemcitabine	Pirimidin antagonist	Diarrhea, occasional nausea, vomiting, mouth sores, poor appetite, watery eyes, sensitivity to light (photophobia), metallic taste in the mouth during the infusion, anemia.	Skin reactions: dryness, cracking, peeling of the skin, darkening of the skin due to hypersensitization to radiation, skin rash, swelling, redness, pain, peeling of the skin on the palms of the hands and the soles of the feet. Hair thinning, nail discoloration, falling of the nails, hand-and-foot syndrome (palmar-plantar erythrodysesthesia).	[40,41,42]
Paclitaxel (Abraxane^®^)	Mitotic block by stabilizing microtubules.	Low blood counts, hair loss, peripheral neuropathy, abnormal ECG, nausea, weakness, fatigue, diarrhea, poor appetite, arthralgias, myalgias, edema, and fever.	Infections, dehydration, constipation, taste changes, skin rash, headache, eye problems, depression, mouth sores, shortness of breath, cough, nose bleeds.	[43,44,45,46,47]
Cisplatin Oxaliplatin	Chelant	Nausea and vomiting. Nausea can last up to 1 week after treatment. Renal toxicity occurs 10 to 20 days after treatment and is usually reversible. Reduction of the concentration of magnesium, calcium, and potassium. Leukopenia and anemia.	Peripheral neuropathy: despite being rare, a serious side effect of decreased sensation and paresthesia can be observed. Sensory loss, numbness and tingling, and difficulty walking can last at least during therapy. These side effects can get progressively worse with treatment. The neurological effects can be irreversible. High frequency deafness. Ringing in the ears. Lack of appetite, alterations in taste, metallic taste. Increased values in blood tests that measure liver function. Hair loss, fever. Cisplatin can also affect fertility.	[48,49]
Irinotecan (Onivyde^®^)	Topoisomerase I inhibitor	Early diarrhea occurs within 24 h of drug administration. It is accompanied by symptoms such as a runny nose, increased salivation, tearing, sweating, erythema, and abdominal cramps. This type of diarrhea can occur during drug administration. Late diarrhea occurs 24 h after drug administration and usually reaches its highest intensity around 11 days after treatment. Dehydration and electrolyte imbalance. Nausea, vomiting, weakness, leukopenia, anemia.	Hair loss, poor appetite, fever, weight loss, constipation, dyspnea, insomnia, cough, headache, dehydration, shaking chills, acne, flatulence, erythema of the face, mouth sores, heartburn, swelling in the feet and ankles.	[50]

**Table 3:** Surface proteins in pancreatic cancer cells that can be recognized by the antibodies or aptamers immobilized over the nanoparticle’s surfaces.

Surface Protein in Pancreatic Cancer ^1^	Relevance	References
TFRC	Transferrin receptors (TFRC) are over expressed in 93% of the pancreatic cells. In 2019, Wu demonstrated that nanoparticles can be targeted to pancreatic cancer cells using an aptamer that binds with transferrin receptor protein 1 also known as CD71.	[51,52]
FC	Folate receptor (FR) is a glycosylphosphatidylinositol expressed in more than the 80% of the pancreatic cancer patients. It has a limited expression in healthy cells.	[51,53]
DR5	DR5 is significantly higher than stage II, III, and IV tumors than in stage I tumors. DR5 is associated with TRAIL resistance.	[54]
LOXL2	Regulates the expression of EMT markers. LOXL2 overexpression correlates with poor prognosis in patients with pancreatic cancer.	[55]
HGF	Modulate multiple cell functions, including proliferation, motility, migration, and invasion.	[56]
PD-1/PDL1	PD-L1 is expressed in PDAC, and its overexpression is associated with a poor prognosis. Previous studies reported divergent tumoral PD-L1 levels, ranging from 12 to 90%.	[57,58,59]
VEGF	An important factor regulating angiogenesis, VEGF, is over expressed in more than 90% of PDACs and correlates with a worse prognosis. Seo et al. demonstrated that 93% of PDAC were positive for VEGF protein.	[60,61,62]
HER2 or ERBB2	HER2 protein expression is associated with decreased survival rate. HER2 is overexpressed in 45% of PDAC.	[63]
EGRF	EGFR is overexpressed in 40–70% of pancreatic cancers. Overexpression is correlated with metastasis to other organs.	[64,65,66]
IGF-IR	Overexpression and excessive activation of IGF-IR are associated with malignant transformation, increment of tumor aggressiveness, and protection from apoptosis. IGF-IR targets 70 to 100% of the core metabolic pathways that are often altered in PDAC pathogenesis.	[67,68]
PSCA	Overexpressed in approximately 60% of pancreatic cancers.	[69]
CD40	CD40 agonists tumor growth suppression and extended survival.	[70,71,72]
GCC	GCC is a transmembrane G protein cell-surface receptor activated by the endogenous hormones guanylin and uroguanylin and bacterial heat-stable enterotoxins that plays a role in regulation of fluid and electrolyte balance. It is highly expressed in colorectal cancer and about 60–70% of pancreatic cancers. It is shown to inhibit the growth-suppressing activity of GCC in pancreatic cancer cell lines and pancreatic-patient-derived xenograft (PDX) models.	[73,74,75]
CA19-9	An attractive therapeutic target for PCAD is carbohydrate antigen 19-9 (CA19-9), known as sialyl Lewis A (sLea). It represents a validated biomarker widely used for diagnostic and prognostic in pancreatic cancer. It is a useful predictor of tumor stage and resectability and response to therapy, and is useful for assessing overall survival. A reduction in CA19-9 is an indicator of treatment benefit.	[76,77]
SLC44A4	Localized in tumor stroma, fibroblasts, and tumor epithelial cells. This protein has been evaluated as a prognostic and predictive biomarker.	[78]

^1^ Surface proteins overexpressed in comparison to healthy cells.

## Data Availability

Not applicable.
